# Psychological Distress and Homesickness Among Sudanese Migrants in the United Arab Emirates

**DOI:** 10.3389/fpsyg.2021.710115

**Published:** 2022-02-10

**Authors:** Abdalla A. R. M. Hamid

**Affiliations:** Department of Clinical Psychology, United Arab Emirates University, Al Ain, United Arab Emirates

**Keywords:** psychological distress, anxiety, depression, homesickness, Sudanese, migrants

## Abstract

Migration is a global phenomenon growing in scope, and it can be associated with negative emotions such as sense of impending loss, fear of the unknown, and anxiety about those left at home. The objective of this exploratory study was to examine psychological distress and homesickness among Sudanese migrants in the United Arab Emirates (UAE). Participants were 1444 Sudanese migrants (*M*_age_ = 40.20; SD = 10.98). The Second Version of the Dundee Relocation Inventory was used to assess homesickness, and the 28-item General Health Questionnaire was used to measure psychological distress, depressive and anxiety symptoms, somatic symptoms, and social dysfunction. The results showed that older age and longer duration of residence in the UAE were associated with lower levels of homesickness, psychological distress, and depressive and anxiety symptoms. Further, homesickness was associated with higher psychological distress, somatic symptoms, and depressive and anxiety symptoms. Women and unemployed migrants had higher psychological distress, somatic symptoms, and depressive and anxiety symptoms compared with men and those employed, respectively. Being unmarried was associated with higher levels of depressive symptoms and homesickness, while those married or divorced/widowed showed lower levels of depressive symptoms and homesickness. It was concluded that there is a need to tackle unemployment among migrants in the UAE and address family reunion issues.

## Introduction

Migration is a global phenomenon that is growing in scope. It is defined as a process of social transition involving the movement of persons from one cultural milieu to another (Bhugra and Jones, 2001; Gong et al., 2011). Since early times, humans have travelled from their culture of origin to other cultures for various reasons, such as teaching, trading, learning, converting, settling, and conquering (Furnham and Bochner, 1982). They also travelled for political, educational (Bhugra and Jones, 2001), and financial betterment (Gong et al., 2011), lack of proper jobs, natural calamities, poverty, family reunion, and conflicts (Wickramasinghe and Wimalaratana, 2016). Labour migration has been associated with highly stressful demands, poor work conditions, and poor psychological and physical well-being (Jamil and Kumar, 2020).

The Gulf countries [United Arab Emirates (UAE), Qatar, Kuwait, Saudi Arabia, Oman, and Bahrain], are among the major regions that attract intonational migrants (Zahid and Alsuwaidan, 2014). There are over 35 million international migrants living in Gulf Counties (International Labour Organization, 2019). The majority of these migrants come from India, then, Pakistan, and other countries (Kronfol et al., 2014). Mental health problems facing migrants in the Gulf countries have received little attention compared to metal health issues encountered by migrants in the United States, Canada, and Europe (Kronfol et al., 2014).

Over 83% (8 million out of 9.6 million) of the UAE population are international migrants (Barbato et al., 2021). A large number of the migrant in the UAE are not highly skilled, do not enjoy job security, not well paid, and are having their families living in their home countries. They experience serious psychological problems such as anxiety symptoms and distress (Al-Maskari et al., 2011).

Sudanese international migration is comparatively new, although some historical small-scale migrations to Egypt, Lebanon, and Greece have been documented (Hassan and El kinani, 2002). The advent of oil production in some Arab Gulf countries resulted in the fast modernisation and development of various material aspects of life and led to enormous economic changes (Khalaf and Alkobaisi, 1999). These developments demanded the importation of labour of various skill levels to compensate for the shortage of workers due to the small Gulf population and the need for technical skills required for modernisation (Khalaf and Alkobaisi, 1999). Hence, increasingly more individuals voluntarily migrate to seek jobs in countries lacking a skilled workforce (Chen and Shaffer, 2017). The oil-rich Arab countries of the Gulf have been the main destination for Sudanese migrants (Abusharaf, 1997).

Many factors may contribute to Sudanese migration, including the economy, political instability, military repression (Abusharaf, 1997), enhanced work conditions in host countries, lack of employment opportunities in Sudan, and personal reasons (Guha, 1977). The decision to migrate is frequently accompanied by fear of the unknown and anxiety about those left at home (Carballo and Mboup, 2005) as well as loss and bereavement (Henry et al., 2009).

In their effort to meet the demands of the host culture and excel in their work, migrants find themselves forced to adjust to new cultural norms, lifestyle, and professional expectations (Guomundsdottir, 2015). This adjustment requires changing old habits and traditions and adopting new ones. The adjustment can be psychological, involving feelings such as well-being and satisfaction, and/or social, involving the ability to fit in the host culture (James et al., 2004). This process may involve exceeding demands for the migrant. Attempts to cope with these demands may fail and result in serious psychological problems (Hassan and El kinani, 2002). Research has shown that geographical transition is stressful and may increase the incidence of physical and mental illness. The effect of geographical transition on an individual is determined to a large extent by the personal importance of the change (Fisher, 1990).

Many theories have aimed to explain migratory phenomena. The interruption and discontinuity perspective postulates that transition creates an interruption in routines and existing lifestyle (Fisher, 1989), which may contribute to the incidence of physical and psychological problems. Migration is regarded as a risk factor increasing the incidence of psychological disorders (Chou, 2007; Rousseau and Frounfelker, 2019). Migrants are believed to be highly susceptible to depression (Fisher, 1990; Zou et al., 2015; Urindwanayo, 2018) and anxiety (Rousseau and Frounfelker, 2019) owing to heightened levels of stress experienced during and after migration, and possibly also before migration (Kerkenaar et al., 2013). Therefore, Sudanese migrants may suffer psychological and social isolation as a result of moving to a new culture. In its early stages, the move to a new culture may be accompanied by feelings of emotional deprivation, homesickness, grief, insomnia, loss of appetite, and anxiety. These negative feelings may worsen if the immigrant faces employment difficulties, housing problems, and fear of uncertainty, and medical help may be sought (Hassan and El kinani, 2002).

Homesickness denotes a state of psychological distress experienced as a result of leaving home and living in an unfamiliar environment (Van Tilburg et al., 1996), and is related to both social and environmental qualities of the person’s home (Morse and Mudgett, 2017). Homesickness is defined as “sadness caused by the longing for one’s home or family during a period of absence” (Morse and Mudgett, 2017, p. 97). It is characterised by strongly missing family and friends, and persistently yearning for home (Thurber and Walton, 2007). Further, homesickness has been frequently associated with diverse somatic illnesses, depression, psychological distress, anxiety, behavioural problems, social problems, helplessness, maladaptive coping, and trouble concentrating on themes unrelated to home (Thurber and Walton, 2007). In addition, Fisher (1989) found that homesickness is consistently associated with preoccupation with family, friends, home, and routines. The experience of homesickness almost certainly results in adjustment illness (Thurber and Walton, 2007), which is characterised by behavioural and emotional difficulties within 3 months of experiencing homesickness (stressor) (Saravanana et al., 2017).

Khawaja and Dempsey (2007) assessed psychological distress in international university students in Australia and found that the most common form of distress was obsessive compulsive disorder followed by interpersonal sensitivity, depression, somatisation, and anxiety. A study of the prevalence of depression and anxiety between immigrants and non-immigrants in Austria showed that 9.9% of all participants had symptoms of depression, 12.0% had symptoms of anxiety disorder, and 6.7% had symptoms of both depression and anxiety over the previous 2 weeks; the prevalence of anxiety disorders was significantly higher among women (13.8%) compared to men (10.0%), but no significant gender difference was found in the prevalence of depression (Kerkenaar et al., 2013).

However, being an immigrant *per se* may not predispose individuals to mental health disorders such as psychological distress, anxiety, depression, or somatic symptoms. Many factors, including demographic characteristics, are believed to play a vital role in the susceptibility to mental health problems among immigrants (Bas-Sarmiento et al., 2017).

A number of studies suggested that age, gender, marital status educational level, employment, and length of stay in the host country may influence the mental health of immigrants. For instance, being married decreases the susceptibility to mental disorder (Lee, 2018; Straiton et al., 2014) while being divorced or single increases it (Chou, 2007; Bas-Sarmiento et al., 2017). However, some studies (e.g., Lee, 2018) found that being divorced is associated with improved mental health. Chou (2007) found that older immigrants and those who stayed longer in Australia were less distressed compared with their counterparts. Older immigrants were also found to be less depressed compared with younger ones (Georgiadou et al., 2018; Cervantes et al., 2019). Being a female immigrant increases the vulnerability to mental illnesses in general (Chou, 2007; Bas-Sarmiento et al., 2017) and anxiety in particular (Georgiadou et al., 2018). Employment was also found to be protective against mental illnesses (Straiton et al., 2014; Lee, 2018), and a lower level of education was associated with higher psychological distress (Chou, 2007) among immigrants.

Bas-Sarmiento et al. (2017) conducted a meta-analysis of 21 studies on mental health among immigrants. The results showed that educational level was not a reliable predictor of mental health problems. While some studies revealed that a high level of education protected against mental disorders among immigrants, other studies indicated that a high level of education was a risk factor for mental illness. This may reflect that immigrants with high levels of education might experience frustration when they are not able to achieve their professional objectives. Nonetheless, Lee (2018) found that education was associated with improved mental health among immigrants.

Studies on the mental health of migrant workers in the Gulf Cooperation Council states are scarce, and little is known about this issue (Kronfol et al., 2014). A search of the databases PsyINFO, Science Direct, PubMed central, and PsyARTICLES identified three articles addressing Sudanese migrants’ mental health in general, but no studies investigated the mental health of Sudanese in the UAE. Hence, it is very important to investigate the mental health of Sudanese migrants in the Gulf region and the factors associated with mental health problems. Understanding the mental health problems experienced by Sudanese migrants working in the UAE will provide researchers with data relevant to this group of migrants and may help professionals and authorities to tackle mental health problems in this population.

This exploratory study was aimed to investigate homesickness and mental health in Sudanese migrants in the UAE. It was expected that participants would differ in homesickness, psychological distress, depressive and anxiety symptoms, somatic symptoms, and social dysfunction in relation to their gender, age, length of stay in the UAE, marital status, employment, and educational level. Further, homesickness was expected to be associated with psychological problems.

## Materials and Methods

### Participants

Participants were 1444 Sudanese migrants in the UAE, who were selected from the population of Sudanese nationals living in the UAE using snowball sampling. Male participants comprised 84% (1213), while female participants comprised 16% (231) of the sample. Participants’ ages ranged between 18 and 72 years (*M*_age_ = 40.2, SD = 10.98). About 72.4% of the participants were married, 24.4% single, 2.1% divorced, and 1.1% widowed; 40.2% of the participants held bachelor’s degrees, 8.3% postgraduate degrees, 26.4% high school certificates, 12.4% completed general secondary school, and 12.7% completed or did not complete primary school. Moreover, 56.6% were employed, and 43.4% unemployed. The number of years in the UAE ranged between less than 1 and 46 years (*M* = 13.64, SD = 9.56).

### Instruments

Three sets of questions were use in this study. The first set assessed demographic and biographic information, and length of stay in the UAE. The second was the Second Version of the Dundee Relocation Inventory (Fisher, 1989), which consists of 26 items to assess homesickness. Cronbach’s alpha for this scale in the current study was 0.73. The third set was the 28-item version of the General Health Questionnaire (GHQ; Goldberg and Williams, 1991), which gauges somatic symptoms, depressive symptoms, anxiety symptoms, and social dysfunction. Items were rated on a 4-point Likert scale from 0 to 3. In the current study, Cronbach’s alpha for the total score of this scale was 0.92. Regarding the GHQ subscales, Cronbach’s alpha was 0.85 for somatic symptoms, 0.86 for depressive symptoms, 0.84 for anxiety symptoms, and 0.78 for social dysfunction.

### Procedure

After receiving the ethical approval, heads of Sudanese communities in different areas of the UAE were approached and asked to provide contact information of some Sudanese nationals in their areas. The identified potential participants were contacted and asked to take part in the study. Each participant was asked to provide contact information about other potential participants, and so on. The questionnaires were handed over to participants together with the consent letter. Participants were asked to read the consent letter and indicate their willingness to participate in the study. They were requested to read the instructions, complete all three sets of questions and return them.

### Ethical Considerations

Ethical approval to conduct this research was issued by the Social Sciences Research Ethics Committee (Ref. No. ERS_2018_5819). Participant signed a consent form and agreed to participate in the study. Importance of the research and its objectives were explained to the participants. They were also informed that participation in the study was voluntary and that they could withdraw from the study at any stage. The confidentiality of the collected data was also stressed.

### Data Analysis

All statistical analyses were done using IBM SPSS 25. First, descriptive statistics was used to describe the characteristics of the sample in terms of frequencies and percentages. Pearson correlation analysis was run to identify significant and non-significant associations between the study variables. Further, *t*-test was used to examine gender and employment status differences in health outcome measures. In addition, analysis of variance (ANOVA) and *post hoc* comparisons using the Bonferroni correction were conducted to test differences in health outcome measures in relation to marital status and educational level.

## Results

### Correlations

Pearson correlation analysis revealed that age and length of residence in the UAE were negatively associated with homesickness, psychological distress, and depressive and anxiety symptoms (Table 1). Length of residence in the UAE was also negatively associated with social dysfunction. Older migrants and those who had been in the UAE for longer periods had significantly lower scores on depressive and anxiety symptoms, homesickness, and psychological distress compared to younger migrants and those who had been in the UAE for shorter periods. In addition, those who had been in UAE for longer reported significantly less social dysfunction compared with those with shorter stays (Table 1). Furthermore, homesickness was positively associated with distress, somatic symptoms, and depressive and anxiety symptoms (Table 1). Homesick migrants reported higher levels of the above-mentioned problems.

**TABLE 1:** Intercorrelations between study variables.

Variable	1	2	3	4	5	6	7
1. Age	–						
2. Length of stay	0.64**	–					
3. Somatic symptoms	0.01	−0.002	–				
4. Depressive symptoms	−0.13**	−0.11**	0.31**	–			
5. Anxiety symptoms	−0.08**	−0.09**	0.66**	0.56**	–		
6. Social dysfunction	−0.04	−0.08**	0.44**	0.31**	0.49**	–	
7. Homesickness	−0.107**	−0.11**	0.09**	0.06*	0.08**	0.02	–
8. Psychological distress	−0.07**	−0.08**	0.82**	0.64**	0.89**	0.72**	0.08**

*p < 0.5; **p < 0.01.

### *t*-Tests

Two tailed *t*-test results revealed significant gender differences in somatic symptoms, depressive and anxiety symptoms, and psychological distress, with migrant women scoring higher than men on these variables (Table 2). No significant gender differences were found in homesickness or social dysfunction.

**TABLE 2:** Gender differences in distress, somatic symptoms, depressive symptoms, anxiety symptoms, social dysfunction, and homesickness.

Variables	Males (*n* = 1213)		Females (*n* = 231)		*T*	*df*	*p*	
	*M*	SD	*M*	SD				Cohen’s *d*
Somatic symptoms	10.76	3.67	12.20	4.67	−5.227	1438	0.001	0.343
Depressive symptoms	7.96	2.31	8.41	2.72	−2.649	1439	0.008	0.178
Anxiety symptoms	10.35	3.69	11.17	4.37	−2.998	1439	0.003	0.203
Social dysfunction	12.00	3.23	11.96	3.17	0.191	1439	0.849	0.012
Homesickness	49.06	5.63	48.64	4.98	1.052	1442	0.293	0.079
Psychological distress	41.05	10.03	43.73	12.22	−3.584	1439	0.001	0.240

The results also showed that employed and unemployed immigrants significantly differed in psychological distress, somatic symptoms, and depressive and anxiety symptoms, with unemployed migrants consistently scoring higher on these variables (Table 3). No significant differences were found in social dysfunction or homesickness according to employment status.

**TABLE 3:** Differences in psychological distress, somatic symptoms, depressive symptoms, anxiety symptoms, social dysfunction, and homesickness in relation to employment/unemployment.

Variable	Employed (*n* = 774)		Unemployed (*n* = 593)		*T*	*df*	*p*	
	*M*	SD	*M*	SD				Cohen’s *d*
Somatic symptoms	10.70	3.49	11.26	4.23	−2.688	1361	0.007	0.144
Depressive symptoms	7.78	1.92	8.29	2.77	−4.056	1362	0.001	0.214
Anxiety symptoms	10.23	3.55	10.81	4.07	−2.799	1362	0.005	0.152
Social dysfunction	11.89	2.91	12.14	3.58	1.421	1362	0.156	0.077
Homesickness	48.87	5.39	49.67	5.71	1.642	1365	0.101	0.144
Psychological distress	40.58	9.15	42.50	11.67	−3.407	1362	0.001	0.183

### Analysis of Variance

The ANOVA showed significant effects of education on homesickness only [*F*(4,1425) = 6.54, *p* < 0.001, partial eta squared = 0.020]. The mean homesickness scores of migrants with primary or no formal education (*M* = 50.80; SD = 5.54) were higher than those of the migrants with high school certificates (*M* = 48.35; SD = 5.47), bachelor degree (*M* = 48.75; SD = 5.09), and postgraduate degrees (*M* = 48.85; SD = 5.02). *Post hoc* comparisons using the Bonferroni correction indicated that individuals with primary school certificate or no formal education experienced the highest levels of homesickness compared with those with high school certificates [mean difference (MD = 2.45; *p* < 0.001), bachelor’s degree (MD = 2.05; *p* < 0.001), and postgraduate degrees (MD = 1.94; *p* < 0.05)].

Further ANOVAs also showed significant effects of marital status on depressive symptoms and homesickness only [*F*(2,1440) = 8.168, *p* < 0.001, partial eta squared = 0.012; *F*(2,1440) = 5.592, *p* < 0.01, partial eta squared = 0.008, respectively]. The mean depressive symptoms score of unmarried participants (*M* = 8.46; SD = 3.32) was significantly higher than that of their married (*M* = 7.90; SD = 1.98) and widowed/divorced counterparts (*M* = 7.56; SD = 1.70). *Post hoc* comparisons using the Bonferroni correction indicated that unmarried migrants were more depressed than married migrants (MD = 0.56; *p* < 0.01). The mean homesickness score of unmarried migrants (*M* = 49.82; SD = 5.72) was significantly higher than that of married migrants (*M* = 48.69; SD = 5.48) but not than that of widowed/divorced migrants (*M* = 49.41; SD = 4.55). *Post hoc* comparisons using the Bonferroni correction showed that unmarried migrants were more homesick than married migrants (MD = 1.12; *p* < 0.01).

## Discussion

The impact of migration on mental health has been the focus of research with different migrant populations. However, studies focusing on the mental health of Sudanese migrants in general, and in the Gulf countries in particular, are scarce. The strength of the current study is its focus on the factors associated with mental health problems among Sudanese migrants in the UAE. The most salient findings of this study indicate that age and length of residence in the UAE were associated with lower levels of psychological distress, homesickness, and depressive and anxiety symptoms among Sudanese migrants. This suggests that getting older or being in the host country for longer may decrease the susceptibility to these psychological problems. The lives of older migrants and those who have stayed for a longer time in the host country may be better established and more secure, which may help them build strong social networks, receive more social support, and develop more adaptive coping techniques to deal with the demands in the host country. These results are in line with Chou’s (2007) findings, which indicated that older immigrants and those who stayed longer in Australia reported lower distress compared with their counterparts. The association between depressive symptoms and young age is also in line with the findings of Georgiadou et al. (2018).

It seems that being married may, somewhat, protect against mental health problems, particularly depressive symptoms and homesickness. Married immigrants who are accompanied by their families in the host country may be less likely to feel lonely and socially excluded. The family provides a comfortable refuge, social support, and a greater chance to establish social relations with other families from their country of origin and other countries. Hence, the level of homesickness and depressive symptoms experienced by married migrants may be less severe compared with those experienced by unmarried migrants. Moreover, the coping resources enjoyed by married migrants may not be attainable for single, widowed, or divorced migrants. This may explain why being unmarried is associated with increased susceptibility to both depressive symptoms and homesickness. This is in line with the findings reported by Straiton et al. (2014), indicating that being married may decrease susceptibility to mental disorders compared with being divorced or single (Chou, 2007; Bas-Sarmiento et al., 2017). The possible influence of marital status on depressive symptoms and homesickness in this study should be treated with caution as effect sizes relating to corresponding results are very small.

As indicated by the association between higher number of years in the UAE and lower level of social dysfunction, being in the host country for longer may protect against social dysfunction. As mentioned above, migrants who reside in the host country for a longer time may establish strong social networks with others and become more acculturated. They may establish social relations with migrants from other countries (Pollock and Van Reken, 2009) or citizens of the host country. This may enable them to function efficiently in the social domain. Alternatively, the ability to function socially may encourage migrants to stay in the host country for longer.

The elevated level of distress, anxiety, and depressive symptoms among homesick migrants reflects the potential effect of homesickness on the mental health of migrants or vice versa. In other words, being homesick makes the migrant more prone to distress, and anxiety and depressive symptoms. Alternatively, being distressed increases homesickness. These results support Palai and Kumar’s (2016) finding of a positive association between psychological distress and homesickness. However, homesickness is considered as a state of distress experienced as a result of leaving home and living in an unfamiliar environment (Van Tilburg et al., 1996). Although some theories postulate that homesickness causes psychological problems (Fisher, 1989), it is not clear which one proceeds the other. Both homesickness and psychological distress, as well as other psychological problems, are health outcome measures. To conclude that homesickness causes psychological distress, longitudinal studies rather than cross-sectional studies are needed to measure homesickness prior to the occurrence of psychological disorders.

The high levels of psychological distress, depressive, somatic, and anxiety symptoms among women suggest that being a migrant woman may increase vulnerability to mental health problems. This can be explained in the light of the well-established literature showing non-psychotic psychological problems to be more common among women compared to men. Further, Sudanese migrant women’s ability to establish social relations may be restricted, especially if they are not employed, which increases their susceptibility to mental health problems. Immigrant women generally lack peer support that may protect them against mental health problems (Urindwanayo, 2018). These results are in line with the findings of Chou (2007) and Bas-Sarmiento et al. (2017) among immigrant samples, but contradict the findings of Palai and Kumar (2016), who found no gender differences in mental health issues among university students. However, the higher rates of somatic symptoms among women support the findings of Wenzel et al. (2005) that women showed higher levels of somatic symptoms compared with men. The higher levels of anxiety symptoms among women are in line with the findings of Kerkenaar et al. (2013) and Georgiadou et al. (2018).

Mental health problems including distress, anxiety, and depressive symptoms, and, to lesser extent, somatic symptoms were alarmingly high among unemployed migrants in this study. Financial difficulties and the inability to meet family needs may put unemployed migrants under immense pressure and consequently result in mental health problems. These problems may become even worse when migrants face unemployment (Hassan and El kinani, 2002). This finding supports the results of McDonald and Kennedy (2006), which indicated that unemployment was associated with poorer psychological health among immigrants.

Similar to previous studies (e.g., Bas-Sarmiento et al., 2017), it seems that educational level may not be a reliable predictor of, or a protective factor against, mental health problems among migrants. Education level was associated only with homesickness, with individuals with primary school education or lower reporting higher levels of homesickness than those with high school certificates, bachelor degrees, and postgraduate degrees, but not those with general secondary school certificates. Individuals with low education level may also have lower incomes. Hence, their ability to bring their families with them to the host country is diminished. This may explain why they are more homesick. While some studies found that a high level of education protects against mental disorders among migrants, other studies indicated that a high level of education is a risk factor for mental illness (e.g., Bas-Sarmiento et al., 2017). However, in the current study higher education was not associated with mental illness. Moreover, partial eta squared valued were very small for differences associated with education in this study, which means that these differences are trivial.

This study implies that mental health of migrants needs to be attended to, as many of them suffer distress, homesickness, and depressive and anxiety symptom. Host countries needs to make mental health services are accessible and affordable for vulnerable migrants, especially women, the young, and the unmarried. Qualified professionals and volunteers may play a certain role in providing psychological assistance and support to needy migrants. The results also imply that resilience to stress and adaptive coping strategies need to be enhanced in migrants. Enabling migrants to bring their families to the host country may provide some sort of support and protection against undue homesickness, distress and other psychological problems. Enhancement of work conditions may also result in positive outcomes.

One of the limitations of this study is its cross-sectional nature. A longitudinal design may help to tackle the predictive limitations of the cross-sectional study and to identify causal relationships between variables. Another limitation is the use of quantitative method, a mixed methods approach using a social constructionist lens may provide a more accurate representation of the experiences of Sudanese migrants. The correlation analysis may be another limitation. It is not clear if the correlations were significant due to the sample size being large or not. Future research may use a deeper analysis to assess how demographic and other variables may predict and explain psychological distress and homesickness. Furthermore, examining personality and coping skills and other confounders may improve our understanding of the factors of mental health problems among Sudanese migrants in the UAE.

## Conclusion

Despite the fact that Sudanese and Emirati cultures are both predominantly Muslim, this study revealed that Sudanese migrants in the UAE experience various mental health problems. Attention should be paid to problems faced by the young productive group of Sudanese migrants. Special measures should be taken to deal with unemployment and family reunion issues, as some young migrants cannot afford to bring their families with them to the host country. Solving such problems will undoubtedly help to reduce migrants’ psychological burden. Preventive measures and early intervention should also be considered in order to lower the morbidity of psychological problems among Sudanese migrants in the UAE. The findings of this study provide valuable information for governments, professionals, and researchers, helping them to understand mental health problems in this group of migrants. Our findings may also contribute to the design of preventive actions and programmes to tackle these problems.

## Data Availability Statement

The original contributions presented in the study are included in the article/supplementary material, further inquiries can be directed to the corresponding author.

## Ethics Statement

The studies involving human participants were reviewed and approved by the Social Sciences Research Ethics Committee (Ref. No. ERS_2018_5819). The patients/participants provided their written informed consent to participate in this study.

## Author Contributions

AH contributed to different stages of this manuscript and agrees to be accountable for the content of the work.

## Conflict of Interest

The author declares that the research was conducted in the absence of any commercial or financial relationships that could be construed as a potential conflict of interest.

## Publisher’s Note

All claims expressed in this article are solely those of the authors and do not necessarily represent those of their affiliated organizations, or those of the publisher, the editors and the reviewers. Any product that may be evaluated in this article, or claim that may be made by its manufacturer, is not guaranteed or endorsed by the publisher.
